# Machine-learning based identification of undiagnosed dementia in primary care: a feasibility study

**DOI:** 10.3399/bjgpopen18X101589

**Published:** 2018-06-13

**Authors:** Emmanuel A Jammeh, Camille, B Carroll, Stephen, W Pearson, Javier Escudero, Athanasios Anastasiou, Peng Zhao, Todd Chenore, John Zajicek, Emmanuel Ifeachor

**Affiliations:** 1 Research Fellow, School of Computing, Electronics and Mathematics, Faculty of Science and Engineering, Plymouth University, Plymouth, UK; 2 Honorary Consultant Neurologist, Faculty of Medicine and Dentistry, University of Plymouth, Plymouth, UK; 3 Consultant in Older Adult Psychiatry, ReCognition Health, Plymouth, UK; 4 Chancellor's Fellow, School of Engineering, Institute for Digital Communications, University of Edinburgh, Edinburgh, UK; 5 Data Scientist, BibInsight, Swansea University Medical School, Swansea, UK; 6 Research Fellow, School of Computing, Electronics and Mathematics, Faculty of Science and Engineering, Plymouth University, Plymouth, UK; 7 Senior Information Specialist, Finance, Contracting and Business Intelligence Directorate, Northern, Eastern and Western Devon Clinical Commissioning Group, Exeter, UK; 8 Professor of Medicine, School of Medicine, Medical & Biological Sciences, University of St Andrews, St Andrews, UK; 9 Research Professor, School of Computing, Electronics and Mathematics, Faculty of Science and Engineering, Plymouth University, Plymouth, UK

**Keywords:** NHS data, primary care, GP practice, machine learning, Read code, dementia

## Abstract

**Background:**

Up to half of patients with dementia may not receive a formal diagnosis, limiting access to appropriate services. It is hypothesised that it may be possible to identify undiagnosed dementia from a profile of symptoms recorded in routine clinical practice.

**Aim:**

The aim of this study is to develop a machine learning-based model that could be used in general practice to detect dementia from routinely collected NHS data. The model would be a useful tool for identifying people who may be living with dementia but have not been formally diagnosed.

**Design & setting:**

The study involved a case-control design and analysis of primary care data routinely collected over a 2-year period. Dementia diagnosed during the study period was compared to no diagnosis of dementia during the same period using pseudonymised routinely collected primary care clinical data.

**Method:**

Routinely collected Read-encoded data were obtained from 18 consenting GP surgeries across Devon, for 26 483 patients aged >65 years. The authors determined Read codes assigned to patients that may contribute to dementia risk. These codes were used as features to train a machine-learning classification model to identify patients that may have underlying dementia.

**Results:**

The model obtained sensitivity and specificity values of 84.47% and 86.67%, respectively.

**Conclusion:**

The results show that routinely collected primary care data may be used to identify undiagnosed dementia. The methodology is promising and, if successfully developed and deployed, may help to increase dementia diagnosis in primary care.

## How this fits in

Improving dementia care through increased and timely diagnosis is a priority, yet almost half of those living with dementia do not receive a timely diagnosis. In England, primary care practitioners are encouraged and given incentives to recognise and record dementia in an effort to improve diagnosis rates. However, dementia diagnosis rates in primary care are still low, and many remain undiagnosed or are diagnosed late, when opportunities for therapy and improving quality of life have passed. This model can automatically identify, from routine data, those patients most at risk of living with undiagnosed dementia. This should help to increase the dementia identification rate in primary care.

## Introduction

Dementia is a progressive neurodegenerative brain disease that results in the death of nerve cells. It severely impairs cognitive function, usually memory initially, resulting in significant disability.^1^ About 856 700 people are living with dementia in the UK, at an annual cost of care of £26 billion.^2^ As life expectancy increases, the number of people in the UK affected by dementia is estimated to be >2 million by 2030, with costs tripling.^3^ A timely diagnosis of dementia is important for ensuring that patients are offered the right treatment and access to services,^4,5^ as well as empowering them to better plan their future, and allow them to access clinical trials. However, dementia diagnosis is complex because it has many types (such as Alzheimer’s disease and vascular dementia),^6^ and the clinical features can overlap with other conditions such as depression. A review of NHS practice suggests that up to 50% of patients may not receive a formal diagnosis of dementia,^7^ which is usually provided by specialist secondary care clinics. GPs have at their disposal several dementia screening tools, such as the Six-item Cognitive Impairment Test, to inform referral to secondary care of patients who present to them. However, patients and carers may ignore memory problems and delay seeking medical help for up to 2.5 years.^8^ Therefore, tools that could automatically identify patients with possible dementia, to facilitate targeted screening, could potentially be very useful and help improve diagnosis rates.

There is strong epidemiological evidence that a number of cardiovascular and lifestyle factors such as hypertension; hypercholesterolaemia; diabetes; obesity; stroke; atrial fibrillation; smoking; and reduced cognitive, physical, or social activities can predict the risk of dementia in later life.^9^ Although work has been done to combine some of these factors to calculate long-term risk scores for dementia,^10–12^ research to predict short-term risk or undiagnosed dementia is limited. Attempts have been made to use primary care data to predict dementia over an 18–54 month interval,^13^ but these are aimed at finding an alternative to the use of biomarkers to predict dementia rather than addressing the issue of under-diagnosis. Work has also been done in the development of dementia risk scores.^10,11^ However, unlike QRISK2^14^ which is used to calculate cardiovascular risk scores, current dementia risk models do not identify patients who may have undiagnosed dementia ^15^ and they require collection of additional data from patients, which limits their use in general practice.^12^ Barnes *et al* ^16^ developed a Dementia Screening Indicator (DSI) using data based on dementia predictors that were identified from four different cohort studies. However, some predictive factors used in developing the DSI model (for example, activities of daily living and mobility) are not routinely collected in primary care.

A machine-learning tool could be used to help identify people likely to have undiagnosed dementia in general practice, for clinical assessment and targeted referral on to memory services, thereby facilitating equality of access to dementia diagnosis and services. This is a priority in the UK,^17^ with likely associated cost savings.

## Method

**Figure 1.**
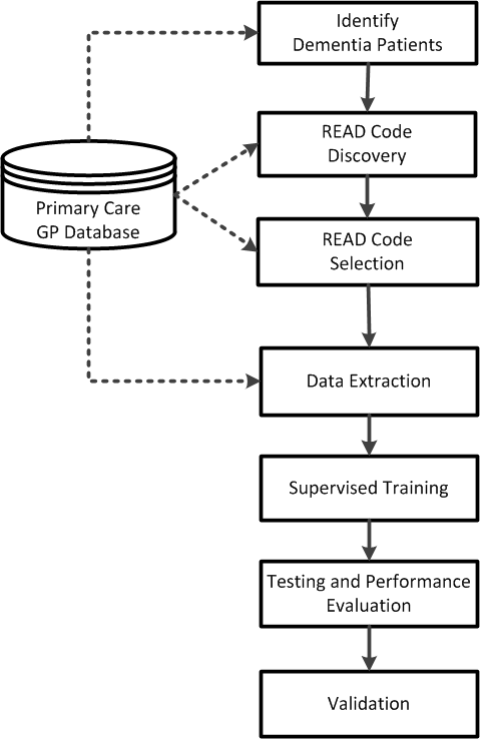
Overview of methodology.

Figure 1 provides an overview of the methodology that was used to identify those that may have undiagnosed dementia from Read-encoded data routinely collected in primary care. Read codes are a thesaurus of clinical terms that are used to summarise clinical and administrative data for general practice in the UK.^17^ All GP practices that participated in this study used the Read coding system.

The method may be summarised as follows:

- A list of Read codes associated with dementia was compiled and used to identify patients with dementia. This was necessary because there was no indicator in the dataset that specifically marked patients diagnosed with dementia.
- The dataset was explored to identify other Read codes that were assigned to the patients with dementia.
- A subset of Read codes was then determined that have a significant association with patients diagnosed with dementia. The subset of Read codes represents features which may be viewed as Read-encoded risk factors for dementia.
- Data were extracted based on the subset of Read codes identified Prince *et al*.^3^
- The extracted dataset was then used to develop a supervised machine learning-based model that is able to identify patients with dementia.
- The performance of the model to identify patients with dementia was tested and evaluated.
- The model’s prediction of the dementia status of patients by GP practices was then validated.

### Data source

NHS Devon (now part of Northern, Eastern and Western Devon Clinical Commissioning Group) had access to data from primary care used in a project to identify patients at risk of unplanned admissions, so that GPs could take preventive action. The primary care data included demographics, long-term conditions, and consultations of patients from 105 participating GP practices from 2010–2012. There were 106 GP practices in NHS Devon; only one did not participate, and that was a small practice that serves a homeless community. It was thus not representative of a standard practice. The large amount of clinical data in the NHS Devon dataset makes it an excellent resource for relevant research on risk factors for dementia and the investigation of undiagnosed cases on a part of the population of the South West of the UK. The practices involved in the project were approached and appropriate approvals were sought. Each practice was sent an email inviting them to consent to their pseudonymised data to be used in this study. Eighteen of 105 practices consented to take part. The data were extracted from the practices that consented.

### Summary of data and participating GP practices

**Figure 2.**
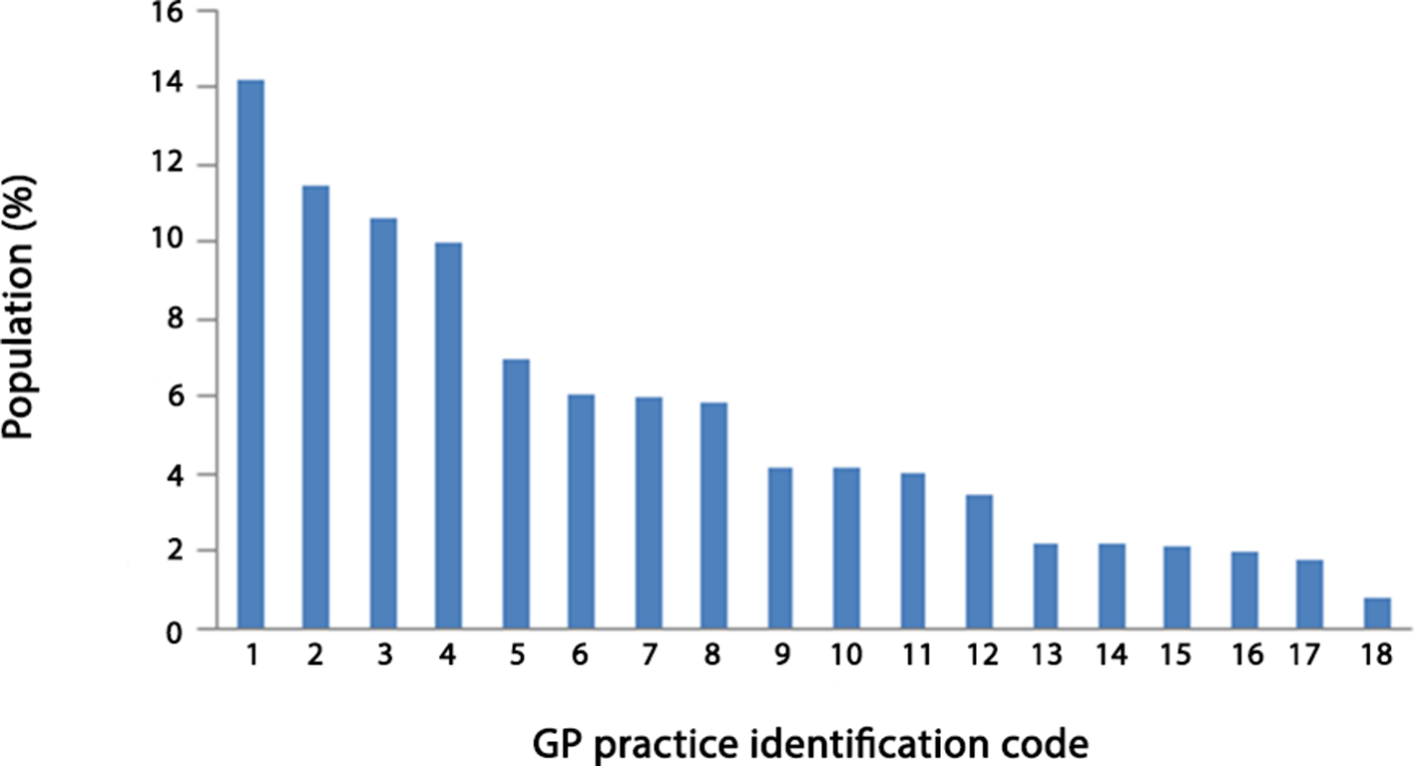
Percentage of study population contributed by each participating GP practice.

Data collected in the period 1 June 2010–1 June 2012 were used. Only data from patients aged >65 years were included. The dataset contains Read codes assigned to patients for each visit to their GP. Patients NHS number was pseudonymised for data protection. There are 26 843 patients and 15 469 Read codes, of which 4301 were diagnosis codes, 5028 process of care codes, and 6101 medication codes. The Read codes are sorted into diagnoses, process of care, and medication chapters.^18^ Diagnosis codes record diagnosis, medication codes record any medication that may have been prescribed, and process of care codes record history, symptoms, examinations, tests, and so on. Figure 2 shows the percentage of the study population that was contributed by each participating GP practice. There is an even sex distribution with 46% male and 54% female patients. In terms of spatial distribution, based solely on the GP surgery that an event was recorded, the majority of the events originated from a small number^5^ of surgeries across Devon.

Of the 18 GP surgeries that participated, there were four city practices, eight town practices, and six smaller rural practices. The four city practices covered one third of the population of patients in the study.

### Identifying patients with dementia

Patients with dementia were identified in the GP dataset using a list of Read codes associated with dementia. The list was compiled from Quality and Outcomes Framework (QoF) codes for dementia,^19^ QoF dementia subset,^20^ other sources,^21^ and by searching the Clinical Terminology Browser, under the guidance of a consultant old-age psychiatrist. Patients who had any of the Read codes in the list assigned to them any time during the study period were assumed to have been diagnosed with dementia.

### Identifying profile of Read codes associated with dementia

The hypothesis of this study is that it should be possible to identify undiagnosed dementia from a profile of Read codes assigned to patients in primary care. The Read codes represent risk factors (such as high blood pressure), symptoms (such as forgetfulness), and behaviours (such as not attending hospital appointments), which are routinely collected in primary care. The authors initially explored the dataset to identify patients with dementia, and identified other Read codes that were also assigned to patients with dementia, apart from those in the dementia list. The features used in the classification are binary and represent the presence or absence of the corresponding Read code in the patient’s data. This disregards how many times the patient may have attended the clinic in relation to a specific problem. Sophisticated feature selection and classification techniques^22^ were used to select the smallest subset of Read codes which capture the complex patterns of Read codes that have a significant association with dementia. Feature selection is often used in machine-learning to select a subset of features that may maximally improve classification performance^23^ and reduce the potential for overfitting.^24^ The feature selection process allowed the identification of a profile of Read codes that may be used to classify dementia and healthy patients with clinically acceptable sensitivity and sensitivity values of at least 80%. Specifically, machine learning-based feature selection algorithms were used to identify a subset of *k* codes that can adequately represent all the other *n* codes assigned to patients with dementia while discarding (*n-k*) codes.^25^ This is based on evaluating the diagnostic value of each individual Read code in classifying patients with dementia and healthy patients.^26^

### Developing a machine learning-based model to identify dementia

Machine-learning was used to derive a classifier to model the different features that characterise patients with dementia so that the derived classifier can be used to detect possible underlying cases of dementia. The Read codes that were selected in the feature selection process were used as features for developing a dementia classification model. The number of times patients were assigned a given code was ignored. This was necessary to ensure that the number of times a patient visits their GP does not influence the determination of their dementia status, thereby making the classifier more generic. The Read codes that were used to determine patients with dementia were removed from the set of features that were used in the classification, as they are related to dementia.

A dataset was extracted from the primary care data around the selected features. The dataset was used to train a machine-learning classifier to learn to discriminate between patients with dementia and healthy patients. The extracted dataset had 850 patients with dementia and 24 858 healthy patients, which represents an imbalance in the size of the two groups. Without any additional procedure, the machine-learning classifiers would be biased towards learning to recognise healthy patients. To compensate for this bias and to emphasise the importance of also learning to recognise patients with dementia, a cost-sensitive classifier^25^ was used. This methodology is based on setting the cost of misclassifying patients with dementia much higher than that of misclassifying healthy patients.

By identifying a profile of Read codes associated with dementia, the authors were able to develop a model that may be able to discriminate between patients with dementia and healthy patients. The University of Waikato (WEKA) open-source toolbox^25^ for developing machine learning-based models for class prediction was used. Support vector machine (SVM),^27^ naïve Bayes (NB),^28^ random forest (RF),^29^ and logistic regression (LR)^30^ algorithms were used with default settings. These algorithms represent the most widely used algorithms in practice.

SVM is a supervised learning method that is widely used for pattern recognition and dementia diagnosis problems^31–33^ due to its ability to learn from data. SVM maps input training data into a higher dimension and separate binary-labelled training data by a decision boundary that is maximally distant from the two classes. It builds a function from the training data so that the function can classify unseen data. SVM is relatively easy to train and it can handle high dimensional data. WEKA implemented Platt’s sequential minimal optimisation algorithm for training SVM classifiers.^34^

The NB classifier is a supervised machine-learning technique that provides a simple approach to represent, use, and learn probabilistic knowledge to classify unseen data. It is based on Bayes theorem and the theorem of total probability. By assuming all features are mutually independent,^9^ NB calculates probabilities of belonging to a class by counting the frequency and combination of features' values in a given training dataset. It is a fast classifier which is not sensitive to redundant features and has found application in dementia diagnosis.^35,36^ For more information, refer to WEKA’s implementation of NB.^28^

RF is an ensemble learning algorithm-based classification method. It uses training data to construct decision trees (DTs), and classify unseen data by combining individual tree decisions. The key feature of RF is the creation of trees that have small randomised differences in characteristics, which improves generalisation performance. RF is particularly suited to high-dimensional data. It has been increasingly used in dementia detection and classification problems.^37,38^ For more information, refer to WEKA's implementation of RF.^29^

LR is a simple machine-learning approach which is widely used as a starting point in binary classification problems and has been used for early diagnosis of dementia.^39^ LR is a statistical technique that predicts the probability of class memberships given a set of feature values.^40^ For more information, refer to WEKA's implementation of the LR classifier.^30^

A *k*-fold cross validation training and testing strategy was implemented,^41^ which is widely used in machine-learning. It is simple to use and universally accepted because it avoids overfitting.^42^ Using this method, the dataset was automatically divided into ten sub-datasets. One was left out of the training process and used for testing, while the remaining sub-datasets were used to train the machine-learning classifier. This was repeated ten times, with a different sub-dataset left out each time, until all sub-datasets were used for training and for testing.

Four criteria were used to assess the performance of the machine-learning classification: sensitivity, specificity, area under the curve (AUC), and accuracy. These performance metrics are generally used in data mining methods for dementia prediction.^43^ After this initial evaluation, the model was run on the entire primary care dataset to determine how many patients could be identified as possibly living with undiagnosed dementia.

## Results

The authors initially identified a profile of possible risk factors from which it may be possible to identify undiagnosed dementia. An analysis was conducted of the distribution of the complete set of Read codes within people diagnosed with dementia and healthy control patients. The findings guided the inclusion of further Read codes in the analyses and selection of other risk factors (further information available from the authors on request).

It is desirable in machine-learning to have the same number of example data in each class. When the number of examples in each class is significantly different, balance can be achieved by using only a subset of the class with the most examples.^44^ In this study, there are 850 patients with dementia and 24 858 healthy patients in the dataset, which represents an imbalance of 1:29 in the size of the two classes. This imbalance was reduced by extracting 2213 randomly selected healthy patients. This subset, together with the 850 patients with dementia, was used to develop classification models to discriminate between patients with dementia and healthy patients. SVM, NB, RF and LR classifiers were investigated.

**Figure 3.**
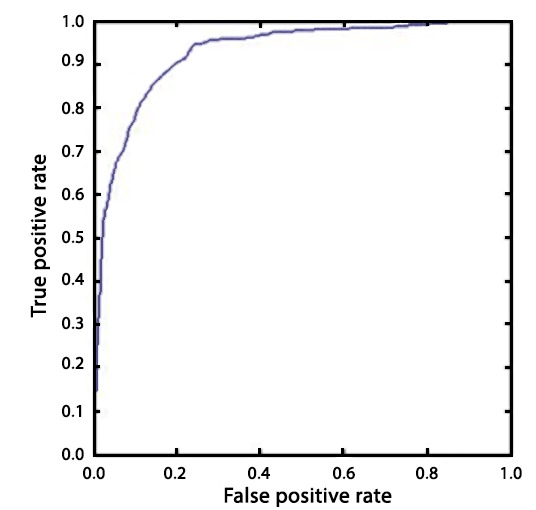
ROC results of the classification.

The performance of the classifiers was assessed, using 10-fold cross-validation, in terms of sensitivity, specificity, accuracy, and AUC. The results showed that the NB classifier gave the best performance with a sensitivity and specificity of 84.47% and 86.67%, respectively (see Table 1). The receiver operating characteristic is shown in Figure 3. With 2213 healthy patients, about 161 may be expected to have dementia (given a prevalence of 7.3%). The model identified 295 patients as possibly having dementia who had not received a diagnosis.

**Table 1.** Naïve Bayes classification results

Performance measure	Result
Sensitivity, %	84.47
Specificity, %	86.67
Correctly classified patients, %	86.06
AUC	0.869

AUC = area under curve.

**Figure 4.**
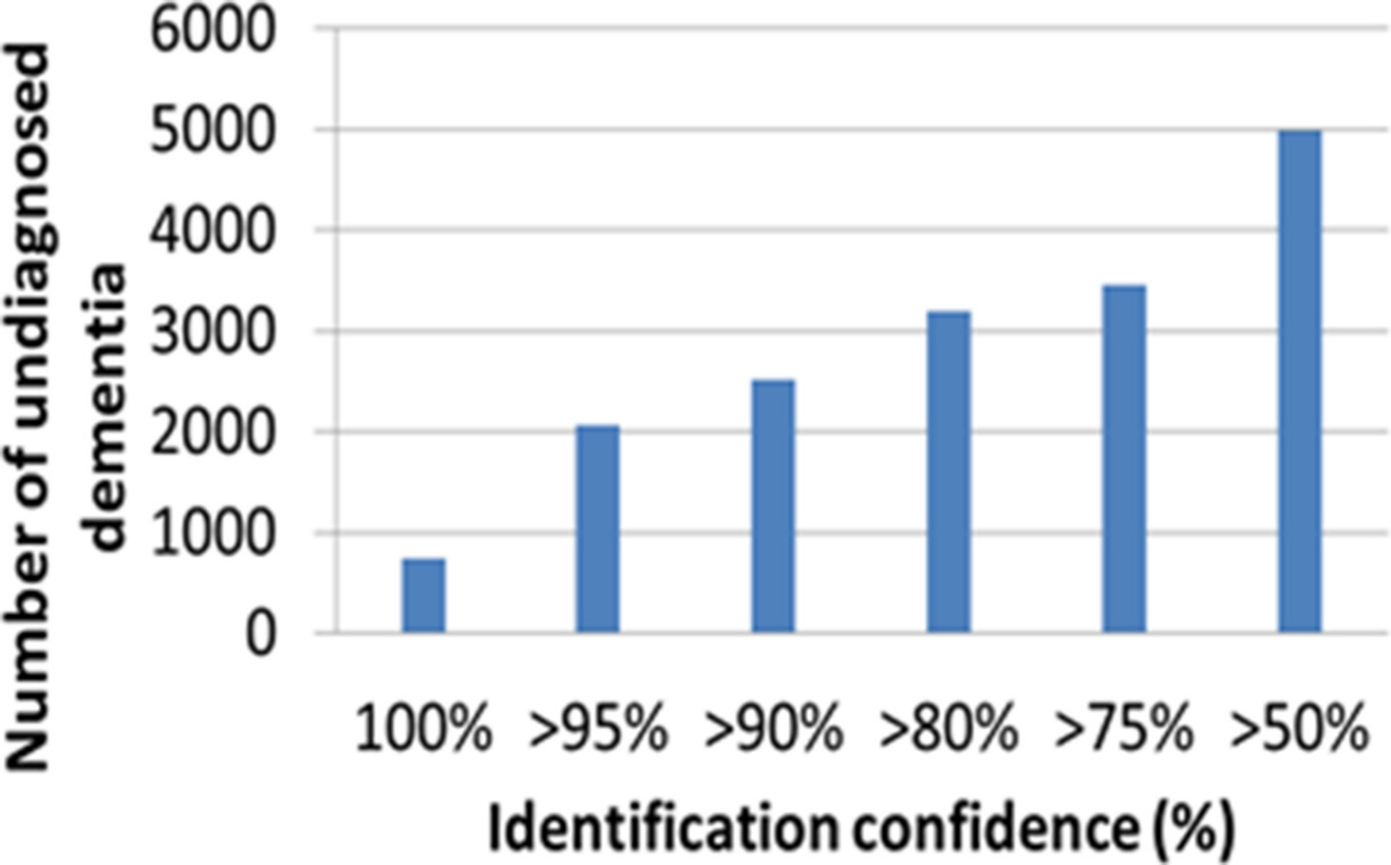
Number of potentially undiagnosed dementia cases.

The performance of the classification model suggests that it can be used at GP practices to facilitate targeted screening by identifying those at risk of undiagnosed dementia. As a proof of concept, the developed model was used to predict undiagnosed dementia in the entire dataset that was shared with the study's authors by GP practices. Figure 4 shows the number of people that this tool identified as living with undiagnosed dementia, based on various thresholds of confidence.

## Validation

To validate these findings, a proportion of true positives (TP), true negatives (TN), false positives (FP), and false negatives (FN) were selected, as classified by the model. The model’s prediction of the dementia status of these patients was sent for validation to GP practices that contributed to the primary care dataset. The validation study was undertaken in July 2015.

Using a lot quality assurance sampling method,^45^ a sample size of 24 each for TP, TN, FP, and FN (making a total sample size of *n* = 96) was calculated to provide sufficient power to allow calculation of overall accuracy levels with a confidence interval of ≤±10%. TP patients are those correctly predicted by the model as having dementia. TN patients have been correctly predicted as not having dementia. FP patients have been predicted by the model as having dementia, but have not had a dementia diagnosis; this group is of particular interest because they might be living with undiagnosed dementia. FN patients have been wrongly predicted by the model as healthy. All 18 GP practices were contacted by telephone or email; 11 responded and agreed to help with the validation. The 11 that responded contributed 21 352 of the 26 843 (79.54%) patients whose data were used in this study. For each of the selected patients, administrative staff was asked to confirm: whether the patient had a diagnosis of dementia; the type of dementia; whether the patient was prescribed dementia medication; date of dementia diagnosis; and dementia codes used. For each of the selected patients, the information received was used to check whether the model’s prediction of a patient’s dementia status was correct. Not all GP practices responded, thus altogether, 19 TP, 14 TN, 21 FP, and 13 FN cases were evaluated by 11 GP practices.

The model predicted 19 subjects to fall in the TP category. Fifteen of these were confirmed in the data to be TP subjects, and the remaining four were confirmed to have dementia by GP surgeries during the validation. However, the validation data showed that these patients were on dementia medication, illustrating the need for more robust validation. The validation showed that the model has a positive dementia prediction accuracy of 78.94%. A negative dementia prediction by the model was confirmed to be the case for 13/14 TN patients. One patient that the model predicted as healthy was confirmed as a dementia patient diagnosed with Alzheimer’s disease. However, the patient was diagnosed with dementia in January 2013, which is outside the study window (June 2010–May 2012).

Five out of 21 patients that the model predicted to have undiagnosed dementia (FP) were confirmed as having dementia by the validation. Three of these were diagnosed with dementia after the study window. This is significant, because they did not receive formal dementia diagnosis within the study window, and were therefore considered not to have dementia. Yet, the tool identified them as potentially living with undiagnosed dementia. Two were diagnosed with dementia during the study window, but were not marked as patients with dementia because they were not assigned a code in the dementia codes list. Yet, they were picked up by the model as patients with dementia because they have similar profile to patients with dementia. The three patients that were predicted to have undiagnosed dementia were diagnosed with dementia 2–30 months after the study period. The remaining 16 patients that the model predicted to have undiagnosed dementia were confirmed to be healthy by the GP practices. These patients were predicted as having dementia by the model because they have similar profiles to patients with dementia, and may therefore benefit from further dementia screening.

The group that was wrongly predicted by the model to have no dementia (that is, the FN group) is not of much interest because these are patients that were known to have been misclassified. The validation was just to confirm that they were patients that had a diagnosis of dementia to start with. Ten patients that the model wrongly predicted as healthy patients were confirmed by GP practices. The model wrongly predicted two patients as healthy, because they were assigned a code from the dementia codes list. One patient had no dementia and was not assigned any code in the dementia codes list.

## Discussion

### Summary

It is generally accepted that a timely diagnosis of dementia has a significant influence on the care, treatment, and quality of life of people who suffer from dementia. Yet dementia diagnosis rates remain low, with up to half of those living with dementia not diagnosed, even in countries with advanced medical care systems.^46^ It has been suggested that screening at GP practices does not result in an increase in diagnosis rates,^47^ and that routine screening is generally not recommended because its efficacy has not been validated.^48^ A cost-effective tool that can be used by GP practices to identify patients likely to be living with dementia, based only on routine data would be extremely useful. Such a tool could be used to select high risk patients who could be invited for targeted screening.

The present authors have developed a machine-learning based classification model that detected undiagnosed dementia, from routinely collected Read-encoded medical history, with sensitivity and specificity values of 84.47% and 86.67%, respectively. The good performance of the model suggests that it could be used at GP practices to facilitate targeted screening to identify those at high risk of having undiagnosed dementia. The model is accurate in identifying undiagnosed dementia, but it also highlights the need for extending the list of dementia codes that are used to identify patients with dementia. The tool has potential incidental advantages; for example, it could be useful in providing greater awareness and understanding of risk factors associated with dementia.

Most diagnoses of dementia, and certainly prescription of dementia medications, would take place either in secondary care or within specialist community memory services. This information would normally be fed back to the GP. For a small proportion of patients managed entirely within primary care, there will be a diagnostic error rate. It is this diagnosis error that a part of this study aims to address, in order to identify to GPs which patients on their caseload have dementia, of which the GP remains unaware

The model needs to be validated before implementation in clinical practice. The authors conducted a limited validation study, whereby a proportion of TP, TF, FP, and FN diagnoses (as classified by the model) were selected from a number of participating GP practices. However, the model requires further and more detailed validation, ideally using large and well-defined clinical cohorts, before it can be used in clinical practice. This would involve the use of datasets, ideally covering different regions of the UK (for example, the Clinical Practice Research Datalink [CPRD] dataset)^49^ to demonstrate robustness and to show that the model can be used in different regions of the UK.

### Strengths and limitations

This is the first demonstration of a machine-learning approach to identifying dementia using routinely collected NHS data. However, this work has some limitations. A list of Read codes based on diagnosis and medication was compiled that represented a diagnosis of dementia. Although guided by clinical input, it is acknowledged that the list that was used to identify patients with dementia may not be exhaustive. It is possible that not all of the patients with dementia in the dataset were identified. It is therefore possible that the 'gold standard' (who in the dataset had dementia and who was healthy) that was used to train the machine-learning classifiers to recognise dementia may not be 100% accurate. This may impact the classification performance.

The dataset was explored to identify other codes assigned to patients with dementia. It was these codes that were used to develop the model. The codes did not also include age, sex, or patient demographics. The inclusion of relevant patient information may improve classification performance further. Additionally, the number of dementia cases in the dataset was relatively small (*n* = 850) compared to the total number of patients (*n* = 26 843). Although this was compensated for by using a cost-sensitive classifier, increasing the number of known dementia cases in the training data may improve performance. The accuracy of all modelling scenarios rests on the quality of the underlying data, which is a potential limitation of this study. To determine the accuracy of coding in the dataset, it would have been ideal to assess the accuracy of diagnostic coding in a sample from each quadrant of the confusion matrix. The resource implications of this made it impractical, but this should be considered in any further evaluation of the model. This tool was based on routinely collected data from 26 843 patients across 18 surgeries in Devon, UK. These data may not be representative of the dementia population of the UK, across patients of different backgrounds and demographics. Data used to develop the model was collected across Devon, and it is therefore possible that it may be specific to the South West of UK. Data collected from across the UK may include a more representative set of Read codes routinely assigned to patients with dementia. Using these data in the development of the model may improve its performance, and possibly make the model more generic for use in primary care across UK.

### Comparison with existing literature

The challenge of improving dementia diagnosis rates provides an opportunity for collaborative research between clinicians and machine learning-based data analysts to develop intelligent data-driven dementia diagnostic models. The use of machine-learning techniques has been used for diagnostic dementia modelling. Pazzani *et al* ^50^ evaluated the potential of machine-learning systems to learn rules for assessing patients based on historical clinical data that was taken from diverse problems, from screening for dementia to the risk of mental retardation. They found that in order for such models to be accepted, they must be consistent with existing medical knowledge. A study by Silva *et al* ^43^ showed that machine-learning classifiers such as neural network (NN) and SVM classifiers can improve dementia classification accuracy. They developed machine-learning classification models based on 10 neuropsychological tests that are commonly used in dementia diagnosis. Their results showed the utility of machine-learning models in the automatic diagnoses of dementia. Williams *et al* ^51^ used neuropsychological and demographic data to train back-propagation NN, SVM, NB, and DT machine-learning techniques to predict Clinical Dementia Rating scores for very mild dementia, MCI, and clinical diagnoses. Williams *et al* showed that machine-learning based modelling can be used to automate clinical diagnoses of dementia. However, they used neuropsychological and demographic data, while the present authors analysed the full set of historical clinical data of a study cohort that was collected over a 2-year period. The machine-learning classification tool in the present study also obtained higher sensitivity and specificity values. Weakely *et al* ^49^ conducted a research study to determine the fewest number of clinical measures that are required for classifying patients with dementia and healthy elderly patients. Their results showed that as few as 2–9 variables may be enough to obtain a clinically useful classification model.

### Implications for research

With the expected growth in dementia prevalence, the number of specialist memory clinics may be insufficient to meet the expected demand for diagnosis.^52^ Furthermore, although current 'gold standards' in dementia diagnosis may be effective, they involve the use of expensive neuroimaging (for example, positron emission tomography scans) and time-consuming neuropsychological assessments which is not ideal for routine screening of dementia. There are several potential research areas that may lead to enhanced performance of this tool. Firstly, healthcare professionals in different regions within the UK may use different Read codes for dementia. A study to identify dementia codes used across the UK will improve the accuracy of identifying those with clinically-diagnosed dementia. Secondly, the tool was based on data collected by 18 GP surgeries in Devon. Using a more nationally representative clinical dataset, such as the CPRD primary care dataset^53^ and the English Longitudinal Study of Ageing dataset, may lead to a tool that could be used across the UK to routinely identify undiagnosed dementia. As future work, the present authors will evaluate the tool more extensively with other datasets, and validate it more extensively at GP practices.
